# Sepsis screening - a cross-sectional study from the Emergency Department Region Hospital Horsens

**DOI:** 10.1186/1757-7241-21-S2-A9

**Published:** 2013-09-09

**Authors:** Nikolaj Raaber, Carsten Brandt, Liselotte Fisker

**Affiliations:** 1 Emergency Department, Region Hospital Horsens, Denmark; 2 Department of Endocrinology University Hospital Aarhus THG, Denmark

## Background

As a part of The Danish Safer Hospital Programme Regional Hospital Horsens introduced the Sepsis package in 2011 in order to reduce unnecessary deaths and harm to patients.

With this study we show how the goals defined in the Sepsis package including 5 elements are met in the Emergency Department Regional Hospital Horsens and how this affects the morbidity of septic patients. At the same time we examined which patients with sepsis who didn’t get screened for sepsis.

## Methods

Study design: Cross sectional study.

We retrospectively reviewed the journals of all patients with af medical condition age >15 years who were admitted to the Emergency Department Regional Hospital Horsens in April 2012.

## Results

After thorough review of data from Opus Electronic Patient Journal we found that almost all patients with sepsis (22 of 29) had received all 5 elements of the Sepsis package (fluid resuscitation, antibiotics, cultivation, lab tests and screening for severe sepsis) but only 12 met all goals after 6 hours (the 6 hour bundle). We found 13 patients (45%) with severe sepsis/septic shock out of which 4 died corresponding to 31% of patients with severe sepsis/septic shock.

## Conclusion

Our data suggests that the implementation of all elements in the Sepsis package are difficult to achieve and that there is place for improval in the Emergency Department Regional Hospital Horsens. To change this a goal directed effort among the staff is needed.

The mortality among septic patients doesn’t seem to have been reduced after implementing the Sepsis package, the number of patients in this study is too small to make any final conclusions.

By enlarging the study to greater number of patients and including a historical control group it will be possible to evaluate the impact of the Sepsis package.

Real time easy registration of observations and treatments are also necessary if better results are to be achieved concerning compliance to all elements of the six hour bundle.

